# Nurse‒physician interprofessional collaboration in the context of expanding nursing practice in primary health care: A comparative qualitative study of Brazil and Germany

**DOI:** 10.1186/s12912-025-02885-5

**Published:** 2025-03-07

**Authors:** Cassiano Mendes Franco, Daria Bula, Beatriz Rosana Gonçalves de Oliveira Toso, Ligia Giovanella, Kerstin Hämel

**Affiliations:** 1 Sergio Arouca National School of Public Health, Oswaldo Cruz Foundation , R. Leopoldo Bulhões, 1480, 21041-210 Rio de Janeiro, Brazil; 2 Faculty of Medicine, Federal University of Rio de Janeiro, R. Laura de Araújo, 36, Rio de Janeiro, 20211-170 Brazil; 3 Department of Health Services Research and Nursing Science, School of Public Health, Bielefeld University, Universitaetsstrasse 25, 33615 Bielefeld, Germany; 4 Western Paraná State University , R. Universitária, Paraná 2069, 85819-110 Cascavel, Brazil; 5 Department of Nursing Science, Faculty of Social Sciences, University of Vienna, Alser Strasse 23/12, Vienna, 1080 Austria

**Keywords:** Interprofessional relations, Physician‒Nurse relations, Patient care team, Nurses, Nurse’s role, Physicians, General practitioners, Primary health care, Qualitative study

## Abstract

**Background:**

The increasing complexity of patients’ health needs has led to the expansion of nursing practices in primary health care (PHC) globally. The corresponding rearrangements of the care process have promoted more horizontal relationships and interprofessional collaboration between nurses and physicians. Our study analyzes the current forms and prospects of nurse‒physician collaboration in the context of expanding nursing practice in PHC in Brazil and Germany.

**Methods:**

We hosted 9 focus groups (4 in Brazil and 5 in Germany) featuring a total of 23 stakeholders who were involved in PHC policy making and 25 practicing nurses and physicians. Brazil and Germany were subjected to comparative analysis using the thematic coding approach suggested by Flick.

**Results:**

Regarding the current forms of nurse‒physician collaboration, focus group participants identified the opportunities and obstacles exhibited by the PHC models employed in their respective countries. In both countries, nurses’ contributions to PHC were associated mainly with the task of meeting complex health needs; however, promoting nurse engagement is challenged by the predominance of physicians’ power in health care policies and practices. With respect to future trends in the expansion of nursing practices in collaboration with physicians, all participants in Brazil supported a complementary approach that focused on increasing the autonomy of nurses in PHC; in Germany, mixed opinions were expressed regarding this issue, with medical stakeholders and some general practitioners (GPs) insisting on a delegation-based approach.

**Conclusions:**

Our study contributes to the literature by highlighting the opportunities and obstacles associated with interprofessional collaboration in the context of expanding nursing practice in PHC. The study highlights the willingness of both nurses and physicians to increase their level of collaboration by encouraging broader nursing practices. However, the power imbalance and hierarchical relations hinder the progress of collaboration between PHC nurses and physicians based on the logic of interprofessionality. The contrasting perspectives, which emphasize an autonomous interprofessional relationship and collaboration based on a subordinate relationship in the context of persistent medical hegemony, reflect certain contextual aspects of these health systems and conceptual approaches to care practices.

## Background

Challenges associated with the epidemiological transition from acute to chronic diseases, the aging population, multimorbidity and a shortage of health professionals are faced by health systems worldwide [1, 2]. Complex health needs can be effectively met by strengthening primary health care (PHC) with the goal of providing accessible and comprehensive health care close to communities and throughout the lifespan of individuals. PHC has proven to be capable of meeting most of the population’s health needs [3, 4]. Nurses contribute to PHC by playing a variety of roles with various functions within the framework of PHC models in different countries [5–7]. In the field of PHC, interprofessional collaboration (IPC) is considered a prerequisite for comprehensive, high-quality care [8, 9].

IPC is defined in terms of integrative cooperation among different health professionals, in which context complementary competencies are combined to deliver the highest quality of care [2, 10]. D’Amour et al. [10] define collaboration through the underlying concepts of sharing, partnership, power, interdependency and process. They refer to IPC as a common space and interdependent relation, where different professionals are challenged to overcome their disciplinary boundaries [10]. Rather than mere plurality or juxtaposition, IPC demands more flexibility in sharing professional responsibilities in a complex system [10]. Interprofessional collaboration is fundamental to all health care settings and particularly important for PHC. According to the Alma-Ata Declaration, PHC has a central role in health systems, with multiple integrative functions [3]. IPC is necessary for PHC to offer promotive, preventive, curative and rehabilitative services because it should integrate all sectors and aspects of national and community development related to health [3]. Notably, PHC relies on a diversity of professionals, including physicians, nurses and others, “suitably trained socially and technically to work as a health team and to respond to the expressed health needs of the community” (p.5) [3]. Recent research has shown that interprofessional collaboration in PHC can enhance its effectiveness, leading to better outcomes in terms of medication management and the care process as well as increasing patient satisfaction; evidence has shown that preexisting and well-defined models of IPC have more benefits [11, 12].

Interprofessional collaboration has been identified as a major driver of innovation in PHC [13]. Whereas PHC provision is still physician-centered in many countries, collaboration with other health professionals, particularly nurses, has intensified, thereby increasing the possibility of new arrangements for patient-centered care and practice [14]. However, in his classical essay ‘the doctor‒nurse game’, Leonard Stein [15] described the notion of subordinated nursing practice. He focused on an interaction in which male doctors are the powerholders who make decisions regarding the care to be provided, while acquiescent female nurses contribute their knowledge regarding patients by covertly guiding physicians to provide effective care. McInnes and colleagues [16], in their review of the facilitators and barriers that impact collaboration and teamwork between general practitioners (GPs) and nurses, concluded that nurses in general practice still often do not participate in the processes of goal setting or decision-making. This exclusion of nurses has been criticized as ineffective [14]. Consequently, previous researchers have called for the promotion of horizontal relationships between these two professions [14, 17].

Horizontal professional relationships are based on the values associated with interprofessional collaboration, i.e., trust, mutual respect, equality, and understanding the other party’s role in the care process [10, 17]. However, the ability of physicians and nurses to develop an understanding of the other profession is hindered if the cooperation between these two parties is episodic, relies on referrals and is characterized by sporadic meetings between these two types of professionals [8, 18].

To address chronic conditions and meet long-term care needs, several countries have increased their efforts to expand nurses’ contributions to PHC [19, 20]. Strengthening the nursing profession has been recognized as a suitable strategy for increasing the effectiveness of approaches to PHC [21, 22]. In addition, some scholars have argued that increasing nurses’ engagement in PHC can help address the shortage of general practitioners, thereby decreasing costs and improving accessibility [23, 24]. Moreover, this strategy can promote progress toward more horizontal relationships between nurses and physicians. The expansion of nurses’ roles is closely related to efforts to implement advanced practice nursing (APN). According to the International Council of Nurses (ICN), advanced practice nurses (APNs) are nurses, such as nurse practitioners and clinical nurse specialists, who have specialized knowledge and typically possess a master’s degree; these nurses play an expanded role in their specific context of practice [25]. In particular, APNs make advanced clinical decisions [25, 26]. In PHC, the actual tasks and responsibilities of APNs vary depending on the country and context in which they work [20]. There are countries, such as the United Kingdom and New Zealand, where APNs are more widespread and assume a broad range of tasks, including authorization for prescriptive tasks [26]. In other countries, including Brazil and Germany, the definition of an APN has not yet been legally established; consequently, protected APN role titles such as nurse practitioners have not yet been established.

One task involved in the implementation of APN is reviewing the distribution of tasks according to the competencies of the relevant health professionals [27, 28]. Task shifting from physicians to other health professionals, including nurses, has been supported with the aim of distributing workload among professionals more evenly, thereby increasing service capacity and reducing health care costs [27, 29]. However, in addition to these promising cost benefits, the process of expanding nurses’ tasks in PHC through, for example, APN aims to enrich PHC services, e.g., by implementing models of care on the basis of chronic disease management [6, 21, 30].

The expansion of nurses’ roles and functions in PHC entails certain changes for all the professions involved, particularly with respect to nurses’ collaboration with physicians. Consequently, this process should be analyzed in terms of its intersections with and meanings for the collaboration of nurses and physicians. In this study, we focus on two countries: Brazil and Germany. Neither country has yet implemented an APN, but its potential to improve access to and quality of PHC has been discussed. On the other hand, Brazil and Germany differ greatly in terms of the manners in which they provide PHC. In Brazil, multiprofessional PHC teams are the standard model of care, a situation that involves task sharing and collaboration between nurses and physicians [31, 32]; in contrast, in Germany, PHC is basically provided by GPs with only marginal integration of nurses [19] and a weak collaboration culture [33].

These similarities and differences between these two countries make it interesting to perform a comparative analysis of nurse‒physician collaboration in the context of the expansion of nursing practice. Comparing these two nations can improve our understanding of how contexts might hinder or promote interprofessional collaboration in potential expansions of nursing practice. In addition, such research can deepen our understanding of the movements toward interprofessional collaboration that the expansion of nursing practice in PHC has promoted in different countries. From the perspective of comparative health systems research, “thinking outside the box” is highly relevant and offers a wide range of suggestions and potential ways of optimizing health systems [34]. Accordingly, this study aims to inform future research and policy regarding how interprofessional collaboration can be shaped by the expansion of nursing practices.

## Methods

### Aim of the study

This study is part of the research project “Strengthening Advanced Nursing Practice and Collaboration in Primary Health Care: Brazil and Germany”. The overarching aim of this research is to identify the possibilities and obstacles associated with APN implementation and the corresponding evolution of interprofessional collaboration in PHC in Brazil and Germany. The following research questions are investigated: (1) How is the collaboration between nurses and physicians in PHC characterized? (2) What perspectives are expressed regarding collaboration between nurses and physicians in the context of expanding nursing practice?

### Study design

A qualitative study design was used to analyze and compare the perspectives of PHC stakeholders and practitioners regarding the current and future collaboration of nurses and physicians in the context of expanding nursing practice in PHC in Brazil and Germany. The study was conducted in accordance with the “Standards for Reporting Qualitative Research” (SRQR) guideline [35]. The approach used was a qualitative cross-country comparative study based on focus groups to obtain a critical understanding of the topic [36]. We used this as a strategy to systematically compare the manifestations of phenomena in different sociocultural environments related to two or more countries [36, 37]. We performed the analysis by means of thematic coding according to Flick [38], a coding approach based on elements of grounded theory. Consistent with the qualitative approach, we conducted researcher triangulation, considering the different backgrounds of the authors [39].

The focus groups guideline was centered on the concepts of interprofessional collaboration [2, 10], PHC [3, 4] and APN [25], as highlighted in the background section. In addition, a review of pertinent literature and documents was previously carried out to understand the meaning of nurses’ professionalization, including the historical and political contexts related to the APN discussion in Brazil and Germany [40]. On the basis of insights and feedback drawn from a pretest, small changes were made. The final guideline was based on five themes, as shown in Table 1.

**Table 1:** Overview of the guideline for the focus group

Theme	Aspects
Establishing the focus of the session: Introduction to the overall topic by the moderator, definition of APN according to the International Council of Nurses, illustration of the development of APN in different countries	
1. Understanding of and experiences with APN	Points of contact and experiences of the participants with APN
2. Collaboration between physicians and nurses in PHC	Current collaboration, needs for improvement, opinions regarding increasing the autonomy of nurses in patient care
3. Strengths, possibilities, and obstacles associated with the implementation of APN	Current tasks of nurses, facilitators and obstacles to the implementation of APN in PHC
4. Role of institutions in the implementation of APN	Management responsibilities, role of professional associations
5. Visions for the future/conclusions	Visions of collaboration and the implementation of APN in 10 years

For each theme, we prepared open-ended questions to obtain a comprehensive overview of the participants’ opinions, in line with the objectives of the overall research project and the premises of the focus group approach [41]. The ICN definition for an APN [25] was introduced to the participants. With respect to interprofessional collaboration, we invited the participants to express their opinions on this topic from the perspective of nurses advancing their practice in PHC, which was not limited to delineated APN roles. Given the comprehensive nature of the focus group guideline and the depth of the discussions, this article focuses specifically on a comparative analysis of the current forms and prospects of collaboration between nurses and physicians in the context of ongoing discussions regarding the expansion of nursing practice in PHC in these two countries. Findings related to the analysis of APN implementation will be addressed in a separate article.

### Research settings: PHC in Brazil and Germany

Brazil has implemented a tax-funded national health system whose public health services are organized and provided by the Unified Health System (SUS, in Portuguese, Sistema Único de Saúde). The health workforce includes 2.4 physicians and 3.5 nurses/1,000 inhabitants, of which 9.7% of physicians and 10.7% of nurses practice in PHC [42, 43, 44]. The Family Health Strategy (FHS) is the main model used for PHC in this context. This model involves teams consisting of 1 physician, 1 nurse, 1–2 nurse technicians and 4–6 community health workers; these teams provide care in PHC centers. The teams are responsible for as many as 4,000 people in a specific geographically defined area and cover 65% of the population [32]. By using a family- and community-oriented approach to PHC, teams work toward health promotion, disease prevention, primary rehabilitation and treatment for most common diseases, and they are expected to coordinate patients within the health system. In addition to individual patient consultations at health centers, physicians and nurses must perform home visits, promote group activities and develop health plans for the population [6, 32].

Nurses are highly involved in the provision of PHC. In addition to same-day appointments, nurses are more engaged in health promotion and disease prevention than physicians are, especially in the context of programs related to mother and child health and chronic conditions. In fact, they play an important role in longitudinal care; most maternal care consultations in PHC, for example, have been the responsibility of nurses in Brazil [45]. Nurses are allowed to diagnose and prescribe in certain conditions (e.g., tuberculosis or urinary infections during pregnancy) following certain rules or protocols (guidelines) that are authorized by health authorities at the municipal, state or federal level. Nurses are usually responsible for team coordination and thus play organizational and management roles [6, 43]. Nurses are educated at universities and studied for a bachelor’s degree over the course of 4–5 years. APN has not yet been implemented in Brazil in accordance with international standards [25]. Debates regarding its implementation are very recent in Brazil, and proposals for master’s degree programs are still being evaluated [46].

For its part, Germany represents the prototype of a social insurance system based on mandatory health insurance [47]. The number of physicians and nurses per thousand habitants in Germany is 4.5 for physicians and 12 for nurses. Approximately 15% (63.372) of practicing physicians are general practitioners [48], and, according to the Federal Statistical Office, 185,000 nurses worked in home care services in 2021 [49]. In contrast to the teamwork-based approach to PHC employed in Brazil, where PHC is provided mainly in public (municipal) PHC centers, PHC in Germany is highly physician-centered and provided mainly in private GP practices. Medical assistants work alongside GPs; these medical assistants are professionally trained and are responsible for administrative tasks and medical procedures under the strict supervision of GPs [19]. In addition, initial attempts have been made to implement an approach involving assistants with a bachelor’s degree with the goal of mitigating the high workloads faced by GPs [19, 50]. As in Brazil, albeit without a community orientation, GPs are responsible for initiating and implementing preventive and rehabilitation measures and treating patients through diagnosis and therapy in a manner that accounts for their family and home environment. GPs are also responsible for coordinating diagnostic, therapeutic and nursing interventions implemented outside their practices (§ 73, Social Code Book V).

In the German PHC context, nurses and GPs do not work as teams to serve a defined population, as is the case in Brazil. Their interactions occur when physicians prescribe home care nursing– which can be regarded an important component of PHC. Patients then choose a home care service provider where nurses assist medical treatment at patients’ homes to avoid hospitalization (§ 37, Social Code Book V). Home care services are also private organizations; only rarely, e.g., in model projects, nurses are part of GP practice teams. In addition, nurses who work in home care services provide long-term care for care-dependent individuals (who are mainly older persons) on the basis of the social long-term care insurance system (Social Code Book XI) [51]. Skilled nurses in Germany usually have three years of vocational training. In addition, a growing number of nurses are obtaining bachelor’s and master’s degrees, although these higher academic pathways remain relatively recent and not yet fully standardized. APN has not yet been implemented in PHC; however, various initiatives, including small-scale model projects aimed at testing APN-based approaches to PHC and master’s programs for APN as well as community health nursing, provide a basis for further progress in this respect [52, 53].

The contrast of the selected countries makes qualitative comparative cross-country study opportune. The Brazilian PHC represents an example of an established model of task sharing and collaboration between physicians and academically educated nurses in PHC centers. In Germany, PHC is GP-centered, with nurses only gradually becoming involved. However, the ongoing academization of nurses provides new opportunities for expanding nursing practices and nurse‒physician collaboration in PHC.

### Field access and data collection

To obtain a comprehensive overview of the current shape of collaboration between physicians and nurses in PHC as well as the prospects for expanding nursing practice through such collaboration, we hosted focus groups in Brazil and Germany that included (a) stakeholders involved in PHC policymaking a1) at the national level and a2) at the regional policy level as well as (b) physicians and nurses practicing in PHC. Considering the participants’ operational knowledge and opinions regarding interprofessional collaboration and APN allowed us to analyze these multifaceted perspectives from various complementary angles, thereby obtaining information regarding the possibilities and requirements of change [54]. At each level (stakeholders and practitioners), we deliberately focused on heterogeneous focus groups that included both physicians and nurses, and we aimed to ensure that both types of professionals were represented equally in all focus groups.

To select the stakeholders, we used a purposive sampling approach. The research team identified key organizations such as health councils, funding bodies and professional associations in both Brazil and Germany at the national and regional levels. We then discussed a suitable and comparable selection for these two countries. At the practitioner level, we chose a convenience sample by following experts’ recommendations and contacted gatekeepers or previous contacts. The participating GPs and nurses were required to have at least two years of work experience in the FHS (Brazil) and in GP practices and/or home care services (Germany), respectively. All the nurses were required to have at least a bachelor’s degree.

In Brazil, the recruitment of practitioners and regional stakeholders focused on the states of Paraná and Rio de Janeiro, as these states have established PHC training programs and exhibit well-defined PHC coordination in the health care network of hub cities; in addition, the research team was located in these two states. In Germany, we did not limit recruitment to specific states; owing to the small number of multi/interprofessional PHC concepts in practice, practitioners and regional stakeholders from nine of the sixteen German states participated in this research.

Focus group participants were contacted via e-mail or telephone and provided with written information regarding the study. In total, nine focus groups including 49 participants were hosted from May 2022 to June 2023 (see Table 2). In each country, we hosted two focus groups involving practitioners and one group involving stakeholders at the federal level; at the regional level, we hosted one focus group in Brazil and two in Germany. In all focus groups, both physicians and nurses were present, except two regional focus groups in Germany, where we conducted two separate focus groups as a result of the time constraints faced by participants; one of these groups involved 2 medical representatives, whereas the other involved 2 representatives from nursing associations. Overall, the number of participants per group ranged from 2 to 9. An overview of the participant characteristics is provided in Table 2.

**Table 2:** Characteristics of the study participants

	Brazil	Germany	Brazil and Germany
**Participants**,** total**	29	19	48
Male	9	6	15
Female	20	13	33
**Age**			
25–34	10	2	12
35–44	14	2	16
>45	4	14	18
n. a.	-	2	2
**Stakeholders**,** total**	**13**	**10**	**23**
Nurses	7	5	12
Physicians	2	3	5
Other professionals	4	2	6
**Practitioners**,** total**	**16**	**9**	**25**
Nurses with a bachelor’s degree	6	2	8
Nurses with bachelor’s and master’s degrees	3	4	7
Physicians	7	3	10

The focus groups were conducted in Portuguese (Brazil) and German and were moderated by trained members of the research group. Other members of the research group recorded the sessions and took field notes. Open questions from the moderator and direct interaction among the participants led to discussions in which the participants shared and commented on their views and opinions [43]. Except one focus group that was hosted face-to-face with practitioners in Brazil, all focus groups were hosted online via Zoom Video Communications^®^ due to the geographical distance among the participants. The sessions lasted between 2 h. and 3.5 h. each; on average, the data collection process took longer in Brazil.

### Data analysis

All focus groups were recorded, transcribed verbatim, anonymized and translated into English, the working language of the research group. The translation process was performed by native speakers of Portuguese or German who also had a very good command of English. To verify the trustworthiness of the translated data, all the transcripts were checked by at least one coauthor. The participants were assigned acronyms for the purpose of anonymization: in the stakeholder focus groups, the acronym SHN was assigned to nurses, SHP to physicians and SHO to members of other professions, whereas in the practitioner focus groups, the acronym RN was assigned to nurses and GP to physicians. Then, numbers were assigned to the participants on the basis of the order in which they spoke, and the corresponding focus group was indicated.

The data were analyzed using thematic coding [38] with the assistance of MAXQDA software (VERBI GmbH). The first step in this process was to address generative and sensitizing questions [38, 55] with the goal of open coding passages that were relevant to the following questions: “Why should nurses and physicians collaborate?”; “Where and how does collaboration currently take place?”; and “What are the conditions associated with interprofessional collaboration and attempts to strengthen nurses’ role in such collaboration?”.

Transcripts from Brazil and Germany were grouped and coded separately to develop the thematic structure of each country. This analysis was conducted through close collaboration between German and Brazilian researchers on the team. In the first step of this process, we summarized excerpts from the nine focus groups on the basis of their meaning in light of the research questions. The statements made by physicians and nurses were carefully observed at this stage to identify contrasts and similarities in their positions. After this open coding step, the codes were organized, synthesized and reviewed inductively to develop the thematic structure, i.e., a structure of themes/categories and subthemes/subcategories, with the goal of answering the questions mentioned above in light of the perspectives of both physicians and nurses as well as stakeholders and practitioners. The emerging codes and themes were discussed and revised several times. The thematic structures of these two countries were then compared to develop a common thematic structure for both countries, which made it possible to highlight the similarities and differences between Brazil and Germany. The final thematic structure (see Table 3) was discussed and revised several times until consensus was reached among the team of authors. Since rich descriptions of the study participants on the topic of interprofessional collaboration enabled us to comprehensively delineate the themes in their context, meanings and relations for each country data saturation was reached.

**Table 3:** Thematic structure comparing perspectives on interprofessional collaboration in PHC of Brazil and Germany

Themes	PHC models shape collaboration	Nurses contribute to meeting health needs	The predominance of physicians’ power	Prospects for interprofessional collaboration
**Brazil**	FHS facilitates coordination of teamwork and shared tasks between nurses and physicians through alternating appointments, joint consultations, etc. Nurses and physicians engage in intensive, direct and easy contact with each other in PHC. FHS encourages interprofessional collaboration.	Nurses’ health-oriented approach to care and contributions to improve access enable them to meet increasingly complex needs	Nurses are still heavily dependent on physicians for clinical tasks; physicians exhibit a paternalistic attitude toward nurses. The need for physicians’ validation of nurses’ decisions disrupts workflow and leads to informal agreements.	Participants support the expansion of nursing practice in interprofessional collaboration based on autonomy and complementarity rather than substitution.
**Germany**	PHC provision focuses on GPs, and multiprofessional teams are generally not used. Nurses and GPs have separate tasks. Nurses provide home care services prescribed by physicians but have poor access to physicians, which could allow them to contribute to care decisions. Interprofessional collaboration is highly limited.	Nurses’ unique insights into the lifeworlds of patients and the possibility of mitigating the workloads faced by physicians contribute to the task of meeting complex health needs	Physicians oversee patients care. Nurses are often seen to be subordinate to physicians. Physicians have control of the funding and regulatory system. Nurses lack representation at the policy level	Participants are divided between those who support the expansion of nursing practice through interprofessional collaboration based on autonomy and complementarity and those who focus on a model of task delegation that does not involve substitution.

By contrasting the characteristics of these countries in terms of the themes and subthemes, we performed a comparative analysis and interpreted them in terms of their contextual meaning while considering the relevant conditions, interactions, and consequences of various phenomena [38, 55]. Owing to financial and time constraints in the research project, we unfortunately could not involve the study participants to check the data and validate the findings of this study.

## Results

### Brazil

#### PHC models shape collaboration

The participating stakeholders and practitioners emphasized the fact that the concept of family health teams entails close cooperation between physicians and nurses as well as a leadership role for nurses. Compared with other health care settings such as hospitals, interprofessional collaboration “*in primary care is easier”*, as “*the professionals already have that profile*,* that worldview*,* those values”* (Brazil-National, SHN1).

In the discussions, the participants referred to close collaboration between PHC nurses and physicians in the context of child and maternal care as well as care for persons with mild chronic conditions on *the basis of “alternating appointments*,* but the responsibility ends up being with the two members of the team itself*” (Brazil-Regional, SHN7). Conducting joint consultations and performing collective activities (e.g., meetings with hypertension patients to provide education and follow-up) as well as community health planning and weekly team meetings were identified by nursing and medical practitioners and stakeholders as leading to relatively easy and direct exchanges between these two types of professionals: “*We exchange information a lot; we share a lot of care*” (Brazil-Practitioners1, RN5). Moreover, nurses and physicians agreed to separate some tasks, such as vaccination and wound management, which are performed exclusively by nurses. However, the oversubscribed population and work overload faced by family health teams lead to isolated approaches in which “*therefore you do not have time to do collaborative work; the work is individual*” (Brazil-Practitioners2, RN4).

#### Nurses’ contributions to meeting health needs

According to the participants in Brazil, the demands and complexity of health needs have increased due to the emergence of chronic diseases and a socially vulnerable population, thereby requiring a more comprehensive approach. They unanimously called for holistic and patient-centered work, as this approach has proven to be effective and responsive regarding contemporary health problems. Thus, “*it is not done with just one professional from the team; we need the whole team to be involved*” (Brazil-Regional, SHO7).

The stakeholders and practitioners reached a general consensus that nurses fundamentally contribute to PHC due to their expanded practice according to the FHS, which is based on high-quality university education; according to them, many physicians in Brazil recognize this fact. In particular, the nursing stakeholders highlighted nurses’ unique vision of a health-oriented rather than disease-oriented care process: “*I think that in the particular context of Brazil*,* thinking about the PHC and its teamwork*,* the nurse is the person who understands the nursing work. […] [This work] involves the social determination of the health-disease process*” (Brazil-National, SHN2).

Given the insufficient supply of PHC physicians in Brazil (which exhibits shortages, high rates of turnover and frequently little experience in PHC) and the greater availability of nurses, participants called for the stronger involvement of nurses in direct patient care to improve access to health care. In this context, some participants (especially GPs) reported that physicians learn from nurses: “*She [the nurse] was my “preceptor” in this context of me being a recent graduate*,* arriving at a family health team without having had any preparation*” (Brazil-Practitioners2, GP3).

#### The predominance of physicians’ power

According to the nurses who participated in this research, the implementation of protocols (guidelines), which is the subject of an ongoing dispute between the professional councils associated with medicine and those pertaining to nursing, is a prerequisite for the expansion of nursing practice, especially regarding prescriptive tasks in the FHS. Medical stakeholders contrasted PHC with hospitals and private practices, where “*nurse prescribing […] sounds like something quite absurd*” (Brazil-National, SHP3), as they perceived these environments to be governed by physician-centeredness, which was quite different from the established approach in the FHS, which is based on task sharing. In addition, the participants (mostly nursing and medical stakeholders) criticized the dominance of physicians in the regulation of health practices in Brazil, which they viewed as even more problematic, as “*unfortunately most of them [physicians] have a market vision only*” and do not acknowledge “*a need for shared care among professionals*” (Brazil-National, SHP5).

Nurses noted that the current protocols, despite their relevance to the process of enabling nurses to prescribe medication in PHC, are often inappropriate because they *“leave gaps*,* questions*,* and uncertainties in conduct”* (Brazil-Practitioners2, RN4). Consequently, most clinical decisions are ultimately made by physicians, indicating that nurses are still highly dependent. Therefore, as a rule, collaboration seems to extend more from nurses to physicians than vice versa. This situation is linked to a rather paternalistic attitude expressed by some of the participating GPs and medical stakeholders, who noted that in their experience, nurses must be assured of their own capacity: “*I always reiterated the importance of nursing to them [nurses]”; “anything you need*,* my door is always open; you can discuss the patients*” (Brazil-National, SHP5).

Because of their limited autonomy in clinical care, nurses referred patients to additional consultations with physicians when their cases did not fit the protocol. “*That’s where the knot is created and collaboration is*,* sort of… Not that it doesn’t happen as it should*,* but there is an impasse*” (Brazil-Practitioners2, RN5). Instead of referring patients, another opportunity involved discussing cases in joint consultations. However, these meetings can both be time-consuming and interrupt the flow of appointments for both physicians and nurses. Furthermore, situations in which nurses demand joint consultations seem to indicate an unnecessary, rather bureaucratic and subservient act pertaining to the validation of procedures in response to unclear or incomplete protocols. As the participants (especially nurses) noted, this situation also leads to informal agreements within the team regarding prescriptions and test requests: “*The nurse ends up making the request outside the medical record*,* which is wrong*,* and this becomes an illegal exercise of medicine. The physician knows what it is*,* he or she stamps it*,* it’s over*,* and the patient goes home*” (Brazil-Practitioners2, RN4).

#### Prospects for interprofessional collaboration

The nursing stakeholders emphasized that a framework is necessary, such as regarding the implementation of APN, which can allow nurses to make decisions in clinical care, including regarding prescriptions, without receiving validation from physicians. From the viewpoint of practitioners, the question of increasing nurses’ autonomy in collaboration with physicians “*cannot be answered individually*” (Brazil-Practitioners2, GP3). Interprofessional collaboration requires a complementary and collective approach: “*What the nurse can offer is different from what I as a physician can offer. […] We collaborate as a category [of workers] when we look at the collective*” (Brazil-Practitioners2, GP3). This claim pertains to not only clinical care but also collective health activities. Both stakeholders and professionals valued the community orientation of the FHS and highlighted the importance of avoiding narrowing collaboration to an individualistic, disease-centered model.

According to nursing stakeholders, during the implementation of APN, nurses must be careful not to “*forget what nursing wants. […] It is the profession of care*,* so we must be careful to not lose our focus*” (Brazil-National, SHN1). They expressed great concern regarding the implementation of APN in the “*attempt to make the nurse fill the vacancy left by the physician”* (Brazil-National, SHN6); this approach would entail that interprofessional collaboration would cease to exist. They raised the concern that “*nurses are not a stopgap*” (Brazil-National, SHN6) and clarified that interprofessional collaboration does not imply that one team can cover a duplicate population. Nurses in the focus groups indicated that physicians should be aware of nurses’ complementary roles “*because the accusation is that the nurse is pretending to be the physician*,* but that’s not it. Nurses are being nurses*” (Brazil-Practitioners2, RN7). GPs and nurses hoped that APN would allow them to work more harmoniously and establish more symmetrical relationships. The focus of APN in the context of interprofessional collaboration should be on the task of improving patient access and connections, “*not doing it with me [nurse] because it is the last option*” (Brazil-Practitioners2, RN7). Therefore, the participants contemplated combining the unique approaches and perspectives of nurses and physicians in an interprofessional way rather than based on a substitution model. With respect to physicians, changes in their roles or identities because of the expansion of nurses’ autonomy and the implementation of new forms of interprofessional collaboration were not significant topics of discussion in the Brazilian focus groups.

### Germany

#### PHC models shape collaboration

According to the focus group participants, interprofessional collaboration between nurses and physicians is the exception rather than the rule in the German health system. Certain fields, such as palliative and geriatric care, feature intensified collaboration, but general PHC relies heavily on physicians. As teams have not yet been implemented in the standard PHC context, “*the only contacts with nursing that you have in general practice are just the home care services or in long-term care facilities*” (Germany-Practitioners2, GP4). Since the activities of nurses and GPs in PHC are performed in different services, they usually do not share tasks. Even if home care nursing is prescribed by physicians, their interaction with nurses is very limited. However, nurses must often contact physicians to obtain prescriptions for their patients, which is typically accomplished via fax, i.e., without a personal meeting, and through the mediation of medical assistants. Nurses indicated that “*unfortunately*,* we are often blocked by medical assistants. Therefore*,* it is very*,* very tedious to have to go through them so frequently*” (Germany-Practitioners1, RN3). Usual communication does not facilitate exchanges and timely responses. The overwhelming workload faced by physicians, according to practitioners, causes them to be “*naturally averse to keeping the communication channels open because it is simply too much. If you truly have to cosign every little bit of crap*” (Germany-Practitioners2, GP3).

The participating nurses were cautious not to generalize a difficult relationship with GPs. They reported examples of direct contact via e-mail or telephone and, less frequently, case discussions; this point was reinforced by GPs and some stakeholders (nursing and medical representatives). Stronger exchanges sometimes occur “*especially when it comes to more difficult cases where you actually meet on site during home visits and discuss things*”, a situation that also enables the GP to “*make better decisions in the prescription or in the treatment. […] But there are also medical practices where this does not work. Where the general practitioner says*,* ‘No*,* I’m not interested in any of that’*” (Germany-Practitioners1, RN4). In this way, nurses’ collaboration is merely a suggestion that may not be accepted.

#### Nurses’ contributions to meeting health needs

German stakeholders and practitioners highlighted the increasing need for community-based rather than hospital-based care. Specifically, they indicated “*that not only one professional group can be responsible for ensuring good and holistic care for these people in primary health care*” (Germany-Practitioners1, RN5). According to some stakeholders and practitioners, the current physician-centered primary care model is limited in terms of its ability to meet complex health needs. Nevertheless, increased nurse involvement in PHC could help meet these complex needs more effectively. Practicing nurses as well as nursing stakeholders highlighted the fact that nurses can complement GPs’ competences because they contribute expertise on the basis of their unique insights into patients’ lifeworlds and social networks; they provide “*a complete overview of the home situation*” (Germany-Practitioners2, RN2). They can address issues of which physicians are not aware or on which they do not have time to work, such as helping patients access social support service networks. In addition, the need for interprofessional collaboration between nurses and physicians was considered evident, especially in areas that lack physicians, such as rural areas, “*because we see that primary health care cannot be guaranteed across the board. That alone is reason enough to say that we need to open our perspective*” (Germany-Practitioners2, GP3).

Unlike the conventional PHC model, participants reported projects across the country in which collaboration between nurses and GPs, on the basis of their particular knowledge, “*actually works well and on an equal footing*” (Germany-Regional1, SHP1). In such model projects, nurses can play a larger role and expand their practice to some degree; some nurses are qualified as APNs, such as community health nurses. The nurses included in the focus groups who participated in model projects reported that they could assess patients’ clinical conditions and the effectiveness of treatment as well as facilitate networking and guidance for patients and families. As they noted, nurses who are involved in such projects are more autonomous, can make more extended home visits with patients than can physicians and have a more comprehensive view that “*is not only from the medical perspective but also always from the nursing care perspective”* (Germany-Practitioners1, RN5). According to one nurse, patients can express some of their needs in the context of this nursing care: *“surprisingly*,* they [patients] tell me that on the very first visit. And that’s not what they tell their general practitioner. So*,* I kind of have a different approach*” (Germany-Practitioners1, RN5). The participants associated the expanded nursing practices facilitated by these projects with improvements in longitudinal care, the effectiveness of medication management, person-centered care, a better work environment and coordination involving interprofessional collaboration.

#### The predominance of physicians’ power

In Germany, although physicians’ representatives can “*grant certain decision-making powers to other professionals*” (Germany-National, SHO1), physicians must oversee patient care. As physicians in general emphasize the fact that they have ultimate responsibility for patients, according to the hegemonic understanding of the medical profession, expanding nursing contributions is possible only through task delegation. Regarding this approach, nurses feel that their contributions are limited by physicians’ predefined prescribing schemes, “*where you must fight to get home visits*,* prescriptions*,* whatever. Where we are also dependent on collaboration with physicians*” (Germany-Practitioners1, RN2).

Nursing stakeholders and practicing nurses criticized that a new way of dividing tasks between physicians and nurses has been hindered by the fact that physicians’ associations are more concerned with a labor market reserve than with improving the care process by adopting an interprofessional approach. These participants felt that nurses had nearly become auxiliary professionals with narrow competences. In particular, the regional medical stakeholders emphasized the fact that nurses can and should extend their roles but only under the delegation of physicians to avoid compromising the quality of care. In line with this position, some GPs mentioned “*the often-low qualifications of colleagues in nursing care*” (Germany-Practitioners2, GP4), which do not allow these nurses to take on and share greater responsibilities.

Some physicians and their representatives viewed this issue less as a question of qualification, instead arguing that nurses, instead of expanding their activities, have limited their own practice; in their observation, nurses seem to fear taking on greater responsibility. Practicing nurses referred to uncertainties regarding medical issues and felt little confidence in their ability to make decisions by themselves: “*But I always think it is difficult when the medication schedule says: ‘Diuretics may be increased’. Okay. So*,* what do we do? How much do we give*,* 50 milligrams of furosemide?*” (Germany-Practitioners1, RN2). The nurses’ discussions in the focus groups also indicated a certain degree of conformity with nurses’ subordination to physicians in practice. For example, they identified nursing as a way of supporting physicians: “*We often got the feedback: ‘It’s good that you’re doing this for us’. So*,* we also support the general practitioners a lot*” (Germany-Practitioners1, RN3). Similarly, both physicians and nurses indicated that the benefit of APN lies in the fact that nurses are “*a relief*,* a big relief*” (Germany-Practitioners1, GP1) for GPs.

Nursing stakeholders, however, criticized the fact that an unbalanced relationship between physicians and nurses is deeply rooted in health politics in Germany. Nurse representatives as well as other nonmedical health professionals are excluded from collective negotiations regarding service provision and funding regarding statutory health insurance. Stakeholders indicated that both health insurers and physicians’ associations are unwilling “*to set up another control mechanism*” (Germany-Regional1, SHP2). Nursing stakeholders partly blame their own profession, as nurses *“keep talking [themselves] down and bashing each other so openly”* (Germany-Regional2, SHN2). Against this backdrop, these participants argued in favor of *“nurses who are able to express themselves in order to push the issue forward”* (Germany-Regional2, SHN3) and *“organized nursing care with a strong voice”* (Germany-Regional2, SHN2).

#### Prospects for interprofessional collaboration

All the participants agreed with the need to promote closer collaboration between GPs and nurses in PHC. While most participants claimed that cooperation between nurses in home care services and GPs should be improved, one GP and some stakeholders, especially nursing representatives, highlighted the notion of multiprofessional teams in health centers or nurses working in GP practices. The participants called for closer nurse‒physician collaboration to ensure more efficient work processes, a better flow of information and more timely decision-making. They recommended regular meetings among professionals, including through videoconferences, better feedback, and the use of shared electronic patient records. With the exception of some medical stakeholders, the participants referred to evidence drawn from international studies indicating “*that competencies can be transferred [from doctors to nurses] and that there is no loss of care*” (Germany-Practitioners2, GP3). All the participants agreed regarding the importance of expanding nursing practices in PHC; however, they envisioned two models of future collaboration to achieve this goal.

In the first model, according to nurse representatives and practitioners (including some GPs), nurses “*want and can do independent work*” (Germany-National, SHN3). The practice of nursing should not entail *“working for the physician but actually also finding a form of care in primary health care that could also work independently of the physician*” (Germany-Practitioners2, GP3). A central concern is patient-centered care, which implies that nurses “*don’t want to compete but simply do the best possible care for the patient*” (Germany-National, SHN3). While “*everyone does their own thing*” (Germany-National, SHN3), one main challenge in this context pertains to interprofessional collaboration: “*where does it all come together?*” (Germany-National, SHN3). The participants argued that nurses have different perspectives on care and therefore complement rather than replace physicians. They adopted a position “*against this [argument of] relieving the physician […] The thing that I expect from an advanced practice nurse in that context is that they provide better interprofessional care. And that’s where each professional group has its specific and own area of responsibility*” (Germany-Practitioners2, GP4). According to practitioners (primarily including GPs), improvements in interprofessional collaboration also require changes in the paradigm that characterize physicians’ understanding of health care and physicians’ roles in PHC, such as their focus on more complex diagnoses and treatments and strengthened gatekeeping.

In contrast, the second prospective model of collaboration, which was described mainly by medical representatives, was drawn from opposing to a framework in which nurses could contribute to prescriptive tasks. These participants argued that “*these [prescriptions] are medical tasks*,* and these are core competencies*”—*“This is also where the final responsibility remains*” (Germany-Regional1, SHP1), namely, with the physician. Physicians were identified as the only actors who are competent in facing the legal and financial consequences of the German health system. Even if nurses are qualified, by analogy, *“it is not enough that one person in the family has the driver’s license*,* and everyone drives a car”* (Germany-National, SHP5). Nurses making decisions currently reserved for physicians would entail *“a transfer of medical treatment”* (Germany-National, SHP5), consequently causing nurses to substitute for and compete with physicians and leading to a “*diffusion of responsibilities*” (Germany-Regional1, SHP1). Medical stakeholders favor a model in which APNs and physician assistants are given autonomy by being identified as two types of professionals who are able to participate in a delegation model under the supervision of physicians: “*A physician assistant can actually take care of part of the physician’s work by delegation*,* not by substitution. […] And nurses could also be involved in this*” (Germany-Regional1, SHP1). For these participants, it was necessary to ensure that “*the overall treatment must always be collaborative; otherwise*,* it won’t work*” (Germany-Regional1, SHP2); however, they also insisted that nurses’ practice must remain linked to physicians’ decisions such that physicians “*make the first prescription*,* which you can also discuss in the long run: that is a model*” (Germany-Regional1, SHP2).

## Discussion

In the focus groups, stakeholders and practitioners discussed the current and future prospects of interprofessional collaboration among nurses and physicians in response to the expansion of nursing practice in PHC, particularly through APN. The study revealed various commonalities and differences in the perspectives of participants from Brazil and Germany.

Regarding their assessment of current forms of collaboration, as shown by the first three themes in the analysis, participants in both countries claimed that collaboration between GPs and nurses is required to meet the complex health needs of the population more effectively, a claim that has been reflected in the literature [8]. Internationally, in PHC, nurses’ participation in health work is characterized by a mix of skills [2, 19]. In both Brazil and Germany, the nurses in the focus groups emphasized their contributions to PHC on the basis of their unique insights into patients’ lives. This point refers to the professional self-concept of nursing as a caring profession, namely, a type of care that is oriented toward patients’ lifeworlds and rooted in their experiences [6]. In this study, in the Brazilian context, the nurses emphasized a social and community-based approach to care, whereas in Germany, a home-based, individual and family-based approach was highly valued as an important contribution of nursing to high-quality PHC. The scope of the nurses’ approach in these two countries is in line with the PHC model employed in their respective contexts, e.g., the community-oriented design of PHC in Brazil, in contrast to the home care model used for nursing in Germany. Nurses’ contributions are particularly important in the context of medicalization, in which increasing numbers of life circumstances correspond to diagnoses and thus lead to the prescription of drugs with the goal of eliminating or mitigating their symptoms [56]. Strengthening PHC from the nurses’ perspective offers the opportunity to ensure that care is not focused on medicalization.

The prospects for future collaboration, especially for the stakeholders who participated in this study, focused on expanding the contributions of nurses to PHC as a means of optimizing health work. A report issued by the Organization for Economic Co-operation and Development (OECD) [4] highlights the excessive and unnecessary concentration of physicians’ responsibility for various tasks in PHC. The involvement of nurses in primary care could thus help relevant actors respond to problems of access due to the difficulty of attracting and retaining physicians in PHC—an issue that, as this study revealed, was more strongly associated with Brazil—and to problems pertaining to work overload among physicians—an issue that was more pronounced in the German context. This situation connects the idea of involving nurses with the goal of addressing physician shortages or mitigating physicians’ excessive workload [57, 58]. However, these arguments are still physician-centric. Instead of involving nurses in collaboration because of their own professional identity, they are actually viewed as convenient and secondary resources to physicians. In this sense, if sufficient physicians were available, these reasons for nurses to collaborate would not apply. Such approaches to the assignment of new tasks and roles to nurses in PHC contradict the idea of exploiting the potential of interprofessional collaboration by, e.g., sharing knowledge and complementary skills [2].

Our study revealed that current forms of collaboration between physicians and nurses in PHC centers in Brazil and Germany are shaped by the predominance of physicians in practical care as well as in health politics. Practitioners in Brazil and Germany have reported that medical associations in their countries impose obstacles to the expansion of nursing practices and the enhancement of interprofessional collaboration with nurses in an attempt to maintain a labor market reserve. Medical power in the political sphere is related to the monopoly of care provision, which guarantees an almost exclusive mandate to physicians regarding diagnoses and prescriptions [59]. In Brazil, this discussion is expressed, for example, in the debate regarding the Medical Act Law [60], and in Germany, it is expressed in the so-called reserved tasks (“Vorbehaltstätigkeiten”) [61]. Kroezen et al. [62] reported that physicians’ jurisdictional control over prescriptions is a common issue worldwide, thus causing nurses to occupy a subordinate position regarding prescriptions in most Western European and Anglo-Saxon countries where they are authorized to perform such tasks.

The power imbalance and hierarchical relationships between physicians and nurses have been recognized as problems that condition the possibility of interprofessional collaboration. However, the approach to PHC, the organization of the health work process and even the sociohistorical structures of each country lead to different arrangements for collaboration. Our research revealed that “the doctor‒nurse game” [15] has different features depending on the context in which it occurs. In our study, a higher level of subordination was evident in Germany, whereas a certain degree of paternalism was observed in Brazil. The study highlighted the fact that even when a team-based PHC model is in place, as in the Brazilian health system, the subordination of nurses can nevertheless occur, albeit in a more covert form. A literature review that included studies from Europe, Oceania and North America also concluded that hierarchical relationships between physicians and nurses persist within teams [16].

This consistently imbalanced power relationship is not related only to professional labor markets. Medical hegemony is ethically supported by the knowledge paradigm that establishes the concepts and competences used to provide health care. The liability assigned to physicians is justified by the dominance of a biomedical paradigm that focuses on pathologies and diagnostic classifications based on medical science [56, 59]. The process of prescription has been disputed by the nursing and medical professions on the basis of knowledge claims aimed at obtaining and securing jurisdictional control, respectively [63]. Such disputes could be observed in the prospects for interprofessional collaboration in both Brazil and Germany when the focus group participants contemplated the development of interprofessional collaboration on the basis of the expansion of nursing practice in PHC. Two models of collaboration were mentioned: one model was based on the complementarity between nursing and medicine and supported increasing the autonomy of nurses in the context of an expanded role in PHC, whereas the other model involved the preservation of the hierarchy between these professions, in which context the reserved medical authority would be responsible for delegating tasks pertaining to the expansion of nursing practice. These results correspond to those reported by Kroezen et al. [63], who highlighted similar opportunities and obstacles regarding the expansion of nursing practice in the Netherlands, which was associated with the knowledge claims made by the nursing and medical professions. As a foundation for collaboration, the model of autonomy rather than delegation involves attributing the ability to make decisions, i.e., to prescribe medication, to professionals other than physicians, which entails the abandonment of exclusivity regarding an extremely valuable instrument in the contemporary knowledge domain, namely, at a time when many life events are medicalized [56]. However, it is also necessary to consider a model of care that is based on a paradigm that ranges beyond prescriptive logic to value relational practices and participatory health promotion, given that care is determined by a health–disease process that encompasses more than biological phenomena. In this sense, all professionals, including physicians, must review their roles and identities in a way that is consistent with a conceptual framework appropriate to the complexity of health care needs.

Our findings suggest a relatively favorable but also cautious stance on the implementation of APN and its implications for interprofessional collaboration in PHC in both Brazil and Germany. Notably, the participants’ understanding of APN varied significantly. These variations were examined in greater detail in the study of the enablers and barriers of APN implementation by Bula et al. (in review). However, the focus group participants expressed concerns about interprofessional collaboration with the expansion of nursing practices. The unanimous endorsement of a collaboration model based on the complementarity of physicians and nurses by the participants from Brazil and, in contrast, the diverging opinions reported by the participants from Germany regarding the benefits of complementarity vs. delegation as a *modi operandi* in physician‒nurse collaboration can be related to the development of nursing in each country. Established university training, protocols/guidelines for autonomous decision-making practices and representations of councils characterize the nursing profession more strongly in Brazil than in Germany [46, 53], which offers better opportunities to expand and strengthen nursing practices in PHC by taking collaboration with physicians into account in an interprofessional rather than multiprofessional way. The rearrangement of responsibilities in PHC was generally identified with task sharing in Brazil, whereas in Germany, it was identified with task transfer. This difference is related to the imprecision of the notion of task-shifting, which is a common expression in the literature, especially that of APN, but which is associated with the notion of transferring or delegating tasks; in contrast, task-sharing has a more explicit meaning that is of greater importance for interprofessional collaboration [28]. However, in both Brazil and Germany, the participants were unanimously opposed to the idea of using nurses as substitutes for physicians, either because doing so could render the nurse’s identity unclear or because it could lead to a situation in which nurses compete with physicians. In the literature, the substitution of physicians by nurses has been widely evaluated, and studies focused on health care outcomes have demonstrated at least similar results regarding the provision of care by both professionals in PHC [64, 65]. In fact, You et al. [66] reported that nurses’ contributions to health indicators can be increased if they are given more autonomy and equality with physicians. Our study reinforces the claim that the aim of expanding nurses’ practice is to strengthen their collaboration by providing them with more autonomy rather than substitution since interprofessional collaboration presupposes that care is provided on the basis of the relationships among different professionals who are characterized by mutual and horizontal ties [2, 10].

In response to contemporary health needs and new care demands, the importance of nurses’ participation in health care, especially in PHC, has increased not only in Brazil and Germany but also in other countries [6, 19]. However, the different challenges faced in this context entail that nurses and physicians can more clearly adopt certain strategic positions depending on the context, e.g., when physicians seek to maintain their centrality in the care process. The prospects for future collaboration varied among the participants in our study because of their different starting points in terms of health system organization and approaches to PHC. These results suggest that collaboration between nurses and physicians is viewed as more interprofessional in Brazil than in Germany. This situation is related to the teamwork experience and the sharing of values for holistic care in PHC in the context of Brazil’s Unified Health System (SUS), especially regarding the Family Health Strategy [67]. In the German health system, on the other hand, intense fragmentation, liberal practice associated with social insurance funding and physician-centered PHC [68] seem to be barriers to attempting to consider the integration of nurses in interprofessional terms rather than identifying it as multiprofessional work. This finding is consistent with Schmid’s [69] observations regarding current financing policies in Germany, which pose challenges for new approaches in PHC due to regulatory structures that largely focus on physicians.

In other words, the different modes of care delivery impact collaboration opportunities between nurses and physicians. The results show that nurses with university training from Brazilian teams in PHC centers provide a consistent framework for interprofessional collaboration between physicians and nurses. Compared with Brazil, in Germany, the findings indicate how the structure of PHC, with its established system of predominantly private GP practices on the one hand and private home care services on the other hand, reflects not only collaboration between physicians and nurses as professionals but also, necessarily, collaboration between these two types of organizations. The chances and obstacles for interprofessional collaboration in general and in the context of the expansion of nursing practice must be reflected against the background of different PHC contexts. The results of our study indicate that nursing professionals in home care services could contribute more strongly to comprehensive PHC in Germany in the future; however, the tasks of nurses in home care services remain limited, as they exclusively provide treatment care (such as administering medication and changing bandages) under the guidance of GPs, in addition to basic personal care and support in domestic care (§ 37, Social Code Book V). The well-established network of home care services in Germany, however, presents significant opportunities for enhancing home-based care for chronically ill individuals, incorporating not only treatment but also promotion, prevention, and rehabilitation services, all of which can be supported by nursing professionals, thereby providing a level of comprehensiveness in PHC that cannot be solely achieved by GPs. The perspective of German PHC provides the first valuable insights into the challenges of integrating advanced nursing contributions into more traditional, physician-centered PHC systems. Until now, medical assistants, and increasingly physician assistants, have been prevalent in GP practices. The roles of physician assistants and medical assistants with additional qualifications have been debated as possible solutions for addressing ongoing shortages in PHC, especially in structurally underserved areas, particularly by younger GPs [70, 71]. This discussion was also partially reflected in our focus groups, where the participating nurses, however, noted that the presence of these assistant roles sometimes posed a barrier to nurses’ effective collaboration with physicians. However, similar to other recent studies (e.g., [72]), physicians demonstrated a willingness to collaborate more closely with nurses to strengthen PHC. In this context, physician representatives showed a positive attitude toward the idea of nurses taking on expanded roles alongside physician assistants and medical assistants. The new Nursing Education Strengthening Act [73], effective in January 2025, incorporates advanced clinical competencies into higher education nursing curricula, focusing on diabetes management, chronic wound care, and dementia. In future studies, the prospects of nurse‒physician collaboration in this changing environment should be investigated in greater detail.

Our study has implications for the development of political conditions and professional education, as they are important prerequisites for the intensification of interprofessional collaboration during the expansion of nursing practice. As a corollary to the results of this research, some of the relevant political conditions entail ethical and legal recognition of the competence of nurses in making clinical decisions without depending on physicians, as other studies have indicated [16, 62]. It is necessary to reach agreements with key players at the political level, such as medical associations and health insurers. A consensus within nursing regarding the development of new forms of collaboration is another such political condition. A relevant prerequisite is professional education, especially with respect to nurses’ broader practice and the joint qualifications of physicians and nurses. These prerequisites are similar to strategies that have been outlined by the World Health Organization since 2010 [2], which focus on the use of political-institutional measures and interprofessional education to implement interprofessional care in health systems.

### Strengths and limitations

Our study has various strengths and limitations with respect to our analysis of the current shapes and prospects of collaboration between nurses and physicians in the context of ongoing discussions regarding the importance of strengthening the role of nurses in PHC in Brazil and Germany. We followed the suggestions of Flick [38] to conduct a comparative analysis of the sociocultural and political backgrounds of these countries, in which context we inductively identified the patterns, opportunities and challenges associated with interprofessional collaboration in the context of strengthening nursing practice.

On the other hand, this study also has several limitations. The characteristics and opinions of the participants included in this research represent a meaningful but nevertheless partial sample of physicians and nurses in Brazil and Germany. Difficulties pertaining to international comparison, such as heterogeneity in terms of research fields and language issues, can be highlighted as challenges for this study. In particular, some caveats should be noted regarding the results for Germany. The separation of the stakeholder focus groups at the regional level between physicians and nurses with a low number of participants could interfere with the results, as participants could elaborate more on their own professions’ point of view than in interprofessional discussion rounds. Many of the statements of the study participants in Germany are related to patients receiving home care, which is a quite selective patient group compared with the broader patient basis of Brazilian nurses. A strength of this study was, however, that some of the participants from Germany were engaged in or had advanced knowledge of model projects which enable nurses to expand their scope of practice. Despite differences in the countries’ samples, it was possible to perceive various directions of dialog among the participants, highlighting parallels during the comparison. The themes and complex relationships between nurses and physicians that emerged in this analysis should be considered in greater depth in future studies.

## Conclusions

This article reports the opinions of stakeholders and practitioners (specifically nurses and physicians) regarding nurse‒physician collaboration in PHC in Brazil and Germany. Few studies have addressed this topic by focusing on attempts to strengthen nursing practices or from an international perspective. While Brazil is an important representative of the Global South and features a national health system and a team-based approach to PHC, Germany is a prominent country in the Global North and features a social health insurance system and an approach to PHC that focuses on general practitioners. These differences, especially in relation to the PHC models implemented in these two countries, determine the appropriate methods of collaboration and future projections regarding interprofessionality between nurses and physicians. Nevertheless, Brazil and Germany seem to be in line with international trends toward strengthening the nursing profession in health work, which must become increasingly collaborative to address contemporary needs.

This paper offers evidence regarding the possibilities associated with changes in the relationship between nurses and physicians. The research highlights the openness exhibited by both nurses and physicians with respect to the intensified integration of nurses into PHC work, especially with respect to decision-making. This process is accompanied by the development of broader concepts pertaining to more patient-centered and comprehensive care. In this sense, beyond the prescription of medications, protocols, and professional jurisdictions, interprofessional collaboration should aim at a counterhegemonic model of care that is more effective and humanistic than the current model, which involves unequal health relationships. To be coherent, such a model must be based on professional autonomy and mutuality rather than simply the replacement of physicians with nurses. Real consensus must be reached regarding notions of collaboration that involve complementarity and delegation, autonomy and authority, task-sharing and task-shifting, and multiprofessionality and interprofessionality. In particular, it is necessary to consider the development of new identities of each profession in the context of a model of care that is suited to the demands of the 21st century.

## Data Availability

The data are available upon request due to privacy-related restrictions. The datasets generated and/or analyzed during the current study are available from the corresponding author upon reasonable request.

## Abbreviations

APN: Advanced Practice Nursing
APNs: Advanced Practice Nurses
FHS: Family Health Strategy
GPs: General practitioners
IPC: Interprofessional collaboration
PHC: Primary health care
SUS: Brazil’s Unified Health System (Sistema Único de Saúde)
